# The Five-Factor Modified Frailty Index as a Predictor of Outcomes in Deep Brain Stimulation Surgery for Parkinson’s Disease

**DOI:** 10.7759/cureus.47547

**Published:** 2023-10-23

**Authors:** Joshua U Hancock, Alexis L Price, Peter G Zaki, Josette C Graves, Katherine C Locke, Trevor Luck

**Affiliations:** 1 Neurosurgery, Drexel University College of Medicine, Wyomissing, USA; 2 Medicine, Drexel University College of Medicine, Philadelphia, USA; 3 Neurological Surgery, University at Buffalo, Buffalo, USA; 4 Orthopedic Surgery, St. Luke's University Health Network, Philadelphia, USA

**Keywords:** post-operative outcomes, frailty, deep brain stimulation (dbs), movement disorders, modified frailty index-5 (mfi-5), parkinson’s disease (pd)

## Abstract

Introduction

Parkinson's disease (PD) is one of the most common neurodegenerative diseases worldwide. Though there are many pharmacological therapeutics approved today for PD, surgical interventions such as deep brain stimulation (DBS) have shown convincing symptom mitigation and minimal complication rates in aggregate. Recently, the concept of frailty - defined as reduced physiologic reserve and function affecting multiple systems throughout the patient - has gained traction as a predictor of short-term postoperative morbidity and mortality. As such, the Modified Frailty Index-5 (mFI-5) is a postoperative morbidity predictor based on five factors and has been used in neurosurgical subspecialties such as tumor, vascular, and spine. Yet, there is minimal literature assessing frailty in the field of functional neurosurgery. With the prevalence of DBS in PD, this study evaluated the mFI-5 as a predictor of postoperative complications in a selected patient population.

Methods

The American College of Surgeons National Surgical Quality Improvement Program 2010-2019 Database was queried for Current Procedural Terminology (CPT) codes, as well as the International Classification of Diseases (ICD)-9 and ICD-10 codes pertaining to DBS procedures in PD patients. Each patient was scored by the mFI-5 protocol and stratified into groups of No Frailty (mFI-5=0), Moderate Frailty (mFI-5=1), and Significant Frailty (mFI-5≥2). The No Frailty group was used as a reference in multivariate and univariate analyses of the groups.

Results

A total of 1,645 subjects were included in the study and were subcategorized into groups of No Frailty (N=877), Moderate Frailty (N=561), and Significant Frailty (N=207) based on their frailty scores. The subjects' mean age was 65.8±9.4 years. Overall, 1,161 (70.6%) were male, while 484 (29.4%) were female. With reference to the No Frailty group in multivariate analysis, patients with moderate frailty experienced greater unplanned readmission (OR 2.613, 95% CI 1.143-5.973, p=0.023), while those with significant frailty experienced greater unplanned readmission (OR 3.723, 95% CI 1.376-10.073, p=0.010), any readmission (OR 2.396, 95% CI 1.098-5.230, p=0.028), non-home discharge (OR 4.317, 95% CI 1.765-10.562, p<0.001), and complications in aggregate (OR 2.211, 95% CI 1.285-3.806, p=0.004).

Conclusions

Until now, the available clinical tools were limited in providing accurate predictions with minimal information for postoperative outcomes in DBS for PD patients. Our data give clinicians insight into the relationship between frailty and surgical outcomes and will assist physicians in preparing for postoperative care by predicting outcomes of significantly frail PD patients receiving DBS therapy.

## Introduction

Parkinson’s disease (PD) is a debilitating, neurodegenerative disorder that is characterized by motor system dysfunction and tremors. Although PD can be treated with a variety of pharmacological interventions, deep brain stimulation (DBS) has been shown to be a safe, non-pharmaceutical, intervention that can drastically augment a patient’s quality of life. Pharmacological interventions are typically attempted in early stage PD, with DBS implantation being performed in refractory PD cases. Follow-up studies on patients after DBS have found a significant improvement in motor system function [1] and survival [2].

Although widely regarded as a safe procedure, DBS is accompanied by a variety of risks that are being evaluated by using predictive models to improve the selectivity of appropriate surgical candidates [3-6]. Recently, frailty scores have been used as predictive models for surgical candidates with some success in a variety of neurosurgical subspecialties, but its value has yet to be adequately investigated in DBS specifically for PD patients [7-9]. These scores are applied to patients with various comorbidities that have been correlated with an increased risk of postoperative complications. Multiple studies evaluating the relationship between frailty and postoperative complications have found that an increased preoperative frailty score is associated with an increased length of stay (LOS), non-home discharge, and increased complication rates within 30 days following surgery [3-6,10,11].

The Canadian Study of Health and Aging Frailty Index (CSHA-FI) encompassed 70 criteria for calculating frailty scores, which was later shortened to the Modified Frailty Index-11 (mFI-11) and then the mFI-5 (Appendices) [12]. These shortened frailty indices have been shown to be appropriate and equivalent for predicting postoperative outcomes [12].

The mFI-5 scoring system includes five categorical variables: history of diabetes, chronic obstructive pulmonary disease (COPD), acute preoperative congestive heart failure (CHF), hypertension (HTN), and dependent functional status [8]. With these five variables, the mFI-5 is a useful tool for clinicians due to the ready availability of its components as part of a standard history and physical examination, and its corresponding ease of calculation. Though there are multiple studies using the mFI-5 to predict outcomes in general surgery [3,6] and neurosurgical specialties such as spine [1], tumor [5], and cerebrovascular [13], its application in predicting DBS postoperative outcomes in PD patients has only been minimally investigated. We hypothesize that increased mFI-5 scores will be associated with poor short-term postoperative outcomes in DBS surgical patients.

## Materials and methods

Study design and population

This study is a retrospective cohort analysis of patient data from the American College of Surgeons National Surgical Quality Improvement Program (ACS-NSQIP) database from 2010 to 2019. This database captures more than 150 clinical variables on individual patient cases, including demographic data, preoperative risk factors and lab values, intra-operative data, and significant events up to postoperative day 30. Given the de-identified nature of this dataset, the current study was cleared under a human subjects’ exemption.

As per previously published inclusion criteria, the ACS-NSQIP 2010-2019 database was queried for Current Procedural Terminology (CPT) codes pertaining to DBS lead insertion (61863, 61864, 61867, 61868) and generator implantation procedures (61885 and 61886), as well as International Classification of Diseases (ICD)-9 (332.0) and ICD-10 (G20.0) diagnosis codes for PD [14]. Patients not diagnosed with PD, and those undergoing cortical lead implantation as well as revision or removal procedures were excluded, all in accordance with previous studies [14,15].

**Figure 1:**
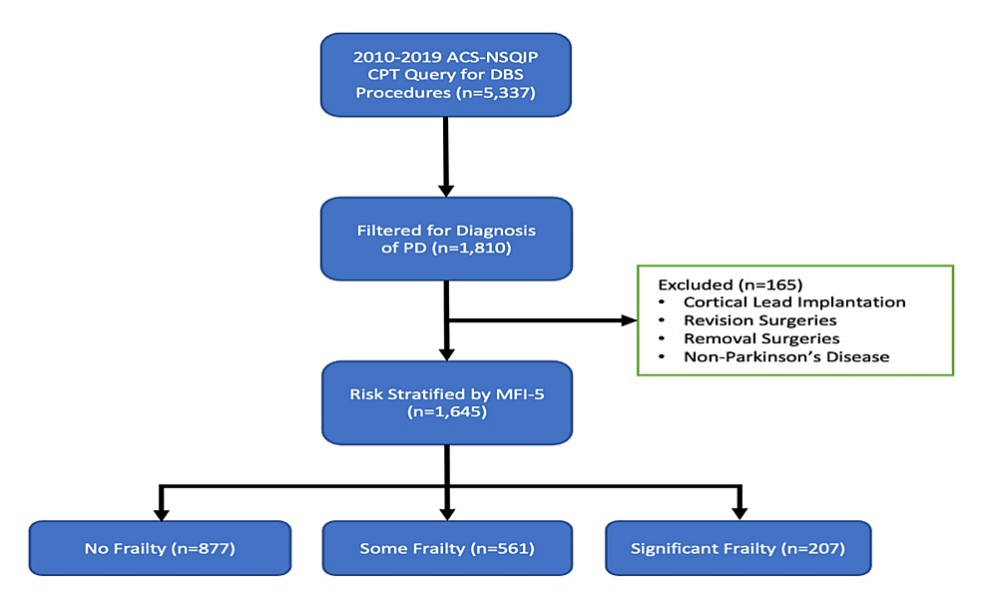
Patient selection.

The mFI-5 scores were calculated for each patient as a sum of the number of conditions present and stratified into No Frailty (mFI-5 = 0), Moderate Frailty (mFI-5 = 1), and Significant Frailty (mFI-5 ≥ 2) groups for analysis. This was done in accordance with previous studies using this index [16-18]. With these parameters, a total sample size of 1,645 subjects was identified (Figure 1).

Outcome measures

Our primary outcomes of interest were any major postoperative event, including systemic complications (such as urinary tract infection, sepsis, pneumonia, unplanned intubation, prolonged postoperative ventilator-assisted respiration >48 hours, cardiac arrest, myocardial infarction, acute kidney injury, deep vein thrombosis, pulmonary embolism, thrombophlebitis, sepsis, septic shock, and death), surgical site infections, and any event necessitating a return to the operating room. Secondary outcomes included readmission rates (any readmission and unplanned readmission, the latter defined by the ACS-NSQIP as a readmission likely due to a postoperative occurrence within 30 days of the principal procedure), rates of non-home discharge, and all complications in aggregate.

Statistical analysis

All calculations and statistics were performed using SPSS Version 26.0 (IBM Corp., Armonk, NY). Categorical variables are expressed with frequencies and proportions, while continuous variables are expressed with means and standard deviations. Univariable analyses were done on patient characteristics, comorbidities, and 30-day outcomes data using the chi-squared test or Fisher’s exact test, as appropriate for categorical variables and the independent samples t-test for continuous variables. The populations compared in this study include No Frailty (mFI-5 score of 0), Moderate Frailty (mFI-5 score of 1), and Significant Frailty (mFI-5 score ≥2). The three groups were analyzed by using the No Frailty group as a reference. Thus, the statistics compare No Frailty versus Moderate Frailty and No Frailty versus Significant Frailty.

On multivariable analysis, characteristics and comorbidities that were significantly different at p<0.05, along with clinically important variables, were gathered for a binary logistic regression analysis to establish independent predictability of the mFI-5 on outcomes. Factors included in this analysis were mFI-5 score, age, sex, body mass index (BMI), smoking status, American Society of Anesthesiologists (ASA) Classification, bleeding disorders, dyspnea, and anemia. Diabetes, COPD, CHF, HTN, and functional status were not included as they are all accounted for by the mFI-5 index. Binary logistic regressions are reported with odds ratio (OR) and 95% confidence intervals (CI). Significance was assessed at p<0.05.

## Results

Patient comorbidities and characteristics

A total of 1,645 patients were identified. Baseline demographic and comorbidity data are summarized in Table 1. Mean age of subjects was 65.8±9.4 years, with 1,161 (70.6%) males and 484 (29.4%) females. The most common demographics were 88.8% White, followed by 2.6% Asian and 1.9% African American. Mean BMI was 27.4±6.1. The three most common mFI-5 criteria were HTN requiring medication (36.2%), followed by diabetes mellitus (11.4%) and dependent functional status (10.8%) (Table 2).

**Table 1:** Patient characteristics. BMI, body mass index; ASA, American Society of Anesthesiologists P-values are with reference to the No Frailty group *Bleeding disorders including chronic anticoagulation. **Anemia according to the WHO criteria of hematocrit <39.0% for males and hematocrit <36.0% for females [7] †Fisher’s exact test

Parameter	All Patients (N=1,645)	No Frailty (N=877)	Moderate Frailty (N=561)	P-value	Significant Frailty (N=207)	P-value
Mean age (years±SD)	65.8±9.4	64.1 ± 9.8	67.3±8.7	<0.001	69.1±7.8	<0.001
Sex				0.038		0.405
Male	1161 (70.6%)	600 (68.4%)	413 (73.6%)		148 (71.5%)	
Female	484 (29.4%)	277 (32.6%)	148 (26.4%)		59 (28.5%)	
Demographics				0.682		0.313
White	1460 (88.8%)	781 (89.1%)	489 (87.2%)		190 (91.8%)	
Black	31 (1.9%)	14 (1.6%)	13 (2.3%)		4 (1.9%)	
Asian	43 (2.6%)	26 (3.0%)	16 (2.9%)		1 (0.5%)	
Native Hawaiian/Pacific Islander	4 (0.2%)	3 (0.3%)	1 (0.2%)		0	
Native American	12 (0.7%)	4 (0.5%)	6 (1.1%)		2 (1.0%)	
Unknown	95 (5.8%)	49 (5.6%)	36 (6.4%)		10 (4.8%)	
Mean BMI (kg/m^2 ^±SD)	27.4±6.1	26.4 ± 5.8	28.0 ± 5.9	<0.001	30.2 ± 6.6	<0.001
BMI (categorical obesity)				<0.001		<0.001
≥30	452 (27.5%)	182 (20.8%)	176 (31.4%)		94 (45.4%)	
<30	1193 (72.5%)	695 (79.2%)	385 (68.6%)		113 (54.6%)	
ASA classification				<0.001		<0.001
1	0	0	0		0	
2	485 (29.5%)	344 (39.2%)	115 (20.5%)		26 (12.6%)	
3	1136 (69.1%)	526 (60.0%)	440 (78.4%)		170 (82.1%)	
4	23 (1.4%)	7 (0.8%)	6 (1.1%)		10 (4.8%)	
Not assigned	1 (0.1%)	0	0		1 (0.5%)	
Smoking (past year)				0.898		0.180
No	1574 (95.7%)	836 (95.3%)	536 (95.5%)		202 (97.6%)	
Yes	71 (4.3%)	41 (4.7%)	25 (4.5%)		5 (2.4%)	
Dyspnea				0.023		<0.001
None	1585 (96.4%)	860 (1.8%)	537 (05.7%)		188 (90.8%)	
Moderate exertional	52 (3.2%)	15 (1.8%)	23 (4.1%)		13 (6.3%)	
At rest	8 (0.5%)	1 (0.1%)	1 (0.2%)		6 (2.9%)	
Bleeding disorders*	17 (1.0%)	3 (0.3%)	11 (2.0%)	0.003	3 (1.4%)	0.088
				0.004†		
Anemia**	271 (16.5%)	115	111	<0.001	45	0.002

**Table 2:** mFI-5 components with the frequency of affected patients.

Parameter	Patients (%) (N = 1,645)
Chronic obstructive pulmonary disease	29 (1.8%)
Congestive heart failure	1 (0.1%)
Diabetes mellitus	188 (11.4%)
Functionally dependent health status	177 (10.8%)
Hypertension	595 (36.2%)

Of the 1,645 subjects, 877 (53.3%) had no frailty, 561 (34.1%) had moderate frailty, and 207 (12.5%) had significant frailty. Compared to the No Frailty group, subjects in both Moderate Frailty and Significant Frailty groups were older (No Frailty: 64.1±9.8 years; Moderate Frailty 67.3±8.7 years, p<0.001; Significant Frailty: 69.1±7.8 years, p<0.001), more likely to be obese (No Frailty: 20.8%; Moderate Frailty: 31.4%, p<0.001; Significant Frailty 45.4%, p<0.001), more likely to have a higher ASA classification (p<0.001 and p<0.001), and more likely to have a history of dyspnea (p=0.023 and p<0.001) and anemia (p<0.001 and p=0.002) (defined according to the WHO criteria of hematocrit <39.0% for males and hematocrit <36.0% for females). Those with moderate, but not significant frailty, were more likely to be male (73.6% versus 68.4%, p=0.038) and to have a history of bleeding disorders (2.0% versus 0.3%, p=0.003).

Univariable analysis

The results of univariable analysis are included in Table 3. Using the No Frailty group as a reference, the Moderate Frailty and Significant Frailty groups had higher rates of both unplanned readmission (3.8% and 4.7% versus 1.4%; p=0.005 and p=0.004) and any readmission (4.6% and 5.8% versus 2.6%; p=0.040 and p=0.020), non-home discharge destination (2.6% and 6.9% versus 1.2%; p=0.050 and p<0.001), and complications in aggregate (8.6% and 13.0% versus 5.8%; p=0.045 and p<0.001). Otherwise, major postoperative complications, individualized systemic complications, wound infection, and reoperation rates had relatively low instances and were not found to be significantly different based on this risk stratification.

**Table 3:** Univariable analysis of outcomes data. DC, discharge; LOS, length of stay; SSI, surgical site infection; ACS-NSQIP, American College of Surgeons National Surgical Quality Improvement Program P-values are with reference to the No Frailty group *Acute renal failure defined as requiring dialysis by ACS-NSQIP **Progressive renal insufficiency defined as >2 rise in creatinine during stay without dialysis ***Prolonged intubation defined as mechanical ventilation >48 hours †Fisher’s exact test

Parameter	All Patients (N=1,645)	No Frailty (N=877)	Moderate Frailty (N=561)	P-value	Significant Frailty (N=207)	P-value	
							Any complication	126 (7.7%)	51 (5.8%)	48 (8.6%)	0.045	27 (13.0%)	<0.001	
Any readmission	61 (3.7%)	23 (2.6%)	26 (4.6%)	0.04	12 (5.8%)	0.02	
Unplanned readmission	39 (2.4%)	11 (1.4%)	19 (3.8%)	0.005	9 (4.7%)	0.004	
Non-home DC	38 (2.3%)	10 (1.2%)	14 (2.6%)	0.05	14 (6.9%)	<0.001	
							Major postoperative complications (systemic insults, reoperation, wound infection)	51 (3.1%)	24 (2.7%)	23 (4.1%)	0.156	4 (1.9%)	0.512	
Myocardial infarction	0	0	0	-	0	-	
Urinary tract infection	6 (0.4%)	2 (0.2%)	2 (0.4%)	0.652	2 (1.0%)	0.115	
Total LOS (days)	0.61±2.37	0.63±2.41	0.51±1.94	0.232	0.77±3.14	0.071	
							Superficial SSI	11 (0.7%)	7 (0.8%)	3 (0.5%)	0.558	1 (0.5%)	0.634	
Deep SSI	0	0	0	-	0	-	
Organ space SSI	2 (0.1%)	0	2 (0.4%)	0.077	0	-	
Wound dehiscence	0	0	0	-	0	-	
Acute renal failure*	0	0	0	-	0	-	
Progressive renal insufficiency**	0	0	0	-	0	-	
Transfusion	0	0	0	-	0	-	
Cardiac arrest	0	0	0	-	0	-	
Clostridium diff colitis	0	0	0	-	0	-	
Deep vein thrombosis	4 (0.2%)	2 (0.2%)	1 (0.2%)	0.84	1 (0.5%)	0.53	
Pneumonia	0	0	0	-	0	-	
Pulmonary embolism	3 (0.2%)	0	2 (0.4%)	0.077	1 (0.5%)	0.039	
						0.191†	
Sepsis	3 (0.2%)	1 (0.1%)	2 (0.4%)	0.326	0	0.627	
Septic shock	0	0	0	-	0	-	
Unplanned reintubation	1 (0.1%)	0	0	-	1 (0.5%)	0.039	
						0.191†	
Prolonged intubation***	0	0	0	-	0	-	
Unplanned reoperation	18 (1.1%)	10 (1.1%)	8 (1.4%)	0.634	0	0.123	
Any reoperation	20 (1.2%)	10 (1.1%)	10 (1.8%)	0.31	0	0.123	
LOS ≥ 3 days	71 (4.3%)	37 (4.2%)	22 (3.9%)	0.782	12 (5.8%)	0.326	
Still in hospital >30 days	1 (0.1%)	1 (0.1%)	0	0.426	0	0.621	
Death	2 (0.1%)	2	0	0.258	0	0.492	

Multivariable analysis

**Figure 2:**
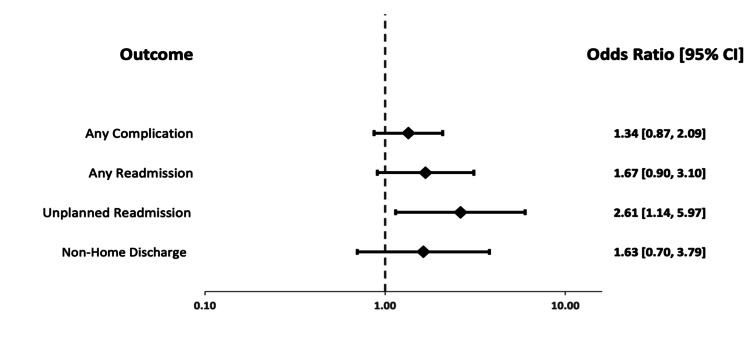
Forest plot of odds ratios from binary logistic regression. Moderate Frailty versus No Frailty as a reference group.

**Figure 3:**
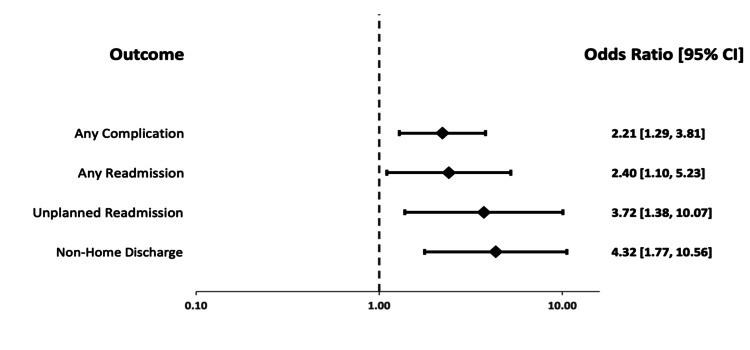
Forest plot of odds ratios from binary logistic regression. Significant Frailty versus No Frailty as a reference group.

The results of multivariable analysis are included in Table 4 and Figures 2, 3. Compared to no frailty as reference, moderate frailty was an independent predictor of unplanned readmission only (OR 2.613, 95% CI 1.143-5.973, p=0.023), while significant frailty remained a significant predictor for all outcomes including unplanned readmission (OR 3.723, 95% CI 1.376-10.073, p=0.01), any readmission (OR 2.396, 95% CI 1.098-5.230, p=0.028), non-home discharge (OR 4.317, 95% CI 1.765-10.562, p<0.001), and complications in aggregate (OR 2.211, 95% CI 1.285-3.806, p=0.004).

**Table 4:** Multivariable analysis of outcomes. OR, odds ratio; CI, confidence interval; DC, discharge; BMI, body mass index; ASA, American Society of Anesthesiologists Controlled for age, BMI, sex, ASA class (1-2 vs. >3), bleeding disorders, dyspnea, anemia, and smoking status.

Outcome	No Frailty	Moderate frailty OR (95% CI)	P-value	Significant Frailty OR (95% CI)	P-value	
						Any complication	Ref	1.344 (0.866-2.085)	0.187	2.211 (1.285-3.806)	0.004		
Any readmission	Ref	1.672 (0.903-3.099)	0.102	2.396 (1.098-5.230)	0.028		
Unplanned readmission	Ref	2.613 (1.143-5.973)	0.023	3.723 (1.376-10.073)	0.01		
Non-home DC	Ref	1.627 (0.699-3.790)	0.259	4.317 (1.765-10.562)	<0.001		

## Discussion

This study is an early evaluation of the mFI-5’s role as a predictor of outcomes for PD patients undergoing DBS treatment and to show its potential application to patient selection for DBS in the future. The limited number of complication events increased the difficulty of achieving significant correlations when attempting to assess the predictive power of the mFI-5 for postoperative issues. Despite this, the mFI-5 still independently predicted multiple outcomes including aggregated complications, readmission rates, and non-home discharge. Given the limitations of what is reported by the ACS-NSQIP database, a direction of future research may include accessing outcome measures that are specific to PD, such as changes in sleep, speech, tremors, balance, and ability to work [14]. Additionally, given the chronicity and progressiveness of PD, future studies should include analysis of postoperative outcomes in follow-up lengths extending to months or even years.

Moreover, this study reaffirms the consensus that DBS procedures are safe with low complication rates. In this study, a mere 7.7% experienced any complication, 3.7% experienced any readmission, and 2.3% experienced non-home discharge. This agrees with a similar study using ACS-NSQIP data for 650 PD patients undergoing DBS procedures, which found that 32 (4.92%) patients experienced major postoperative complications [19]. Of the 650 PD patients, 481 (74%) had thorough discharge information, and of these 481 patients, 3.7% were discharged to a facility and 3.3% had an unplanned readmission. Furthermore, the patients in this comparable study recorded low event rates of pulmonary embolism (0.15%), stroke/cerebrovascular episodes (0.31%), deep venous thrombosis (0.46%), and urinary tract infection (0.77%) [19], while our study recorded these events 0.2%, 0.5%, 0.2%, and 0.4% of the time, respectively.

A recent similar study by Owodunni et al. compared the utility of the risk analysis index (RAI) to the mFI-5 for predicting Clavien-Dindo complications in DBS procedures with data sourced from the ACS-NSQIP database [20]. Their study concluded that RAI was a more robust scale to use for predicting postoperative outcomes in patients undergoing DBS. However, the length of this scale must be acknowledged, considering it requires 14 variables, while the mFI-5 only uses five variables. Of note, their retrospective study investigated DBS implantation in patients with no specific diagnosis, while our study selected DBS implantation in patients with PD. Additionally, their study’s investigation of the mFI-5 only found significant correlations (p<0.001) with frail patients being discharged to non-home facilities (skilled nursing or rehabilitation) [20], while our study found significant correlations (p<0.05) with overall complications, readmission rates, and non-home discharge in severely frail patients. The combined results of these two studies further support the potential utility of the mFI-5 in clinical risk assessment, though limitations are present and supplementary refinement of the scale may be indicated.

Limitations

Conducting a retrospective cohort study using an external database does have its advantages and limitations. The ACS-NSQIP contains a large sample size of DBS patients with adequate data spanning a decade (2010-2019, for our study) on short-term complications after surgery. It is a national dataset compiling information from numerous hospitals, which limits the risk of regional or local confounders and selection biases. These factors allow robust comparisons of outcomes among subgroups of interest for any given procedure recorded by the database.

The ACS-NSQIP’s limitations and disadvantages, however, cannot go unmentioned. Firstly, past medical and surgical histories are insufficiently characterized by the database. Common elements of the medical history, such as hypertension and diabetes, are recorded in a binary fashion, limiting the potential for evaluation of condition severity. Additionally, patients’ list of medications is limited to items impacting bleeding risk, coagulopathy, and wound healing, but tells us nothing about neurological therapeutics. Previous DBS device implantation and the presence of other devices are not recorded. Complete DBS device implantation is commonly performed in two separate procedures, but these data are also not recorded in the database, which obscures the postoperative short-term complication timeline.

Secondly, outcomes of this database, as intended, focus on general surgical outcomes rather than procedure-specific outcomes. The lack of specific neurosurgical measures relating to PD symptoms limits our ability to analyze the practicality of the mFI-5 in predicting short-term outcomes in DBS patients. Lastly, socioeconomic and insurance status are considerable metrics to evaluate concerning discharge destination and readmission [14,19], but these data are not collected. Though there are many limitations present, the ACS-NSQIP provides a consolidated and nationally recognized dataset for statistical evaluation of surgical procedures and has been used previously to report DBS outcomes [14].

Finally, while mFI-5 may be a quick and easy tool, it is arguably reductive and perhaps loses the overall picture of frailty that the CSHA-FI intended. For example, by mFI-5 assessment, a patient who has well-controlled hypertension and well-controlled diabetes would be considered frailer than a patient with completely dependent functional status and no other elements of mFI-5. Indeed, this may raise uncertainty as to who truly has less physiological reserve according to this index. This is not to invalidate the mFI-5 as to where it currently stands but rather to suggest that a more robust version of this index ought to also weigh conditions in terms of severity. This would still allow for simplicity of the index, given that there would still be just a handful of items that could be entered by clinicians into a calculator such as MDcalc [21] or automatically calculated by an electronic medical record software, as is already done with other weighted risk scores such as the atherosclerotic cardiovascular risk score [22].

## Conclusions

Increased frailty was positively associated with independent predictability of the mFI-5 for postoperative outcomes, including major postoperative complications, any readmission, unplanned readmission, and non-home discharge. By exploring the relationship between a patient’s frailty status and postoperative outcomes, surgeons can better predict the postoperative care required for each individual patient. Our paper provided evidence of the potential for the mFI-5 to be utilized as a quick, streamlined tool for physicians to assess and stratify a PD patient’s risk of short-term postoperative complications from DBS, especially in severely frail patients. However, the limitations of the binary data collection methods must be considered and may suggest that a more refined scale is necessary for clinical use. Future studies with long-term follow-up and PD-specific outcomes will assist physicians in predicting necessary clinical care for significantly frail patients who wish to undergo DBS treatment.

## Appendices

**Table 5:** Historical elements of frailty indices, such as CSHA-FI, mFI-11, and mFI-5. CSHA-FI , Canadian Study of Health and Aging Frailty Index; mFI-5, 5-item modified frailty index; mFI-11, 11-item modified frailty index

CSHA-FI	mFI-11	mFI-5
Changes in everyday activities	Chronic obstructive pulmonary disease	Chronic obstructive pulmonary disease
Mood problems	Congestive heart failure	Congestive heart failure
Seizures, partial complex	Diabetes mellitus	Diabetes mellitus
Head and neck problems	Functionally dependent health status	Functionally dependent health status
Feeling sad, blue, depressed	Hypertension	Hypertension
Seizures, generalized	History of angina	
Poor muscle tone in the neck	Impaired sensorium	
History of depressed mood	Peripheral vascular disease	
Syncope or blackouts	Prior myocardial infarction	
Bradykinesia, facial tiredness all the time	Stroke with neurologic deficit	
Headache	Stroke with no residual deficit	
Problems getting dressed		
Depression (clinical impression)		
Cerebrovascular problems		
Problems with bathing		
Sleep changes		
History of stroke		
Problems carrying out personal grooming		
Restlessness		
History of diabetes mellitus		
Urinary incontinence		
Memory changes		
Arterial hypertension		
Toileting problems		
Short-term memory impairment		
Peripheral pulses		
Bulk difficulties		
Long-term memory impairment		
Cardiac problems		
Rectal problems		
Changes in general mental functioning		
Myocardial infarction		
Gastrointestinal problems		
Onset of cognitive symptoms		
Arrhythmia		
Problems cooking		
Clouding or delirium		
Congestive heart failure		
Sucking problems		
Paranoid features		
Lung problems		
Problems going out alone		
History relevant to cognitive impairment		
Respiratory problems		
Impaired mobility		
History of thyroid disease		
Musculoskeletal problems		
Family history relevant to cognitive impairment		
Thyroid problems		
Bradykinesia of the limbs		
Skin problems		
Impaired vibration		
Poor muscle tone in limbs		
Malignant disease		
Tremor at rest		
Poor limb coordination		
Breast problems		
Postural tremor		
Poor coordination, trunk		
Abdominal problems		
Intention tremor		
Poor standing posture		
Presence of snout reflex		
History of Parkinson's disease		
Irregular gait pattern		
Presence of palmomental reflex		
Falls		
Family history of degenerative disease		
Other medical history		
